# Cardiac magnetic resonance imaging parameters as surrogate endpoints in clinical trials of acute myocardial infarction

**DOI:** 10.1186/1745-6215-12-204

**Published:** 2011-09-14

**Authors:** Steffen Desch, Ingo Eitel, Suzanne de Waha, Georg Fuernau, Philipp Lurz, Matthias Gutberlet, Gerhard Schuler, Holger Thiele

**Affiliations:** 1 University of Leipzig - Heart Center, Department of Internal Medicine/Cardiology, Leipzig, Germany; 2 University of Leipzig - Heart Center, Department of Diagnostic and Interventional Radiology, Leipzig, Germany

**Keywords:** Myocardial infarction, surrogate endpoints, cardiac magnetic resonance imaging, validity, reliability, clinical trial

## Abstract

Cardiac magnetic resonance (CMR) offers a variety of parameters potentially suited as surrogate endpoints in clinical trials of acute myocardial infarction such as infarct size, myocardial salvage, microvascular obstruction or left ventricular volumes and ejection fraction. The present article reviews each of these parameters with regard to the pathophysiological basis, practical aspects, validity, reliability and its relative value (strengths and limitations) as compared to competitive modalities. Randomized controlled trials of acute myocardial infarction which have used CMR parameters as a primary endpoint are presented.

## Introduction

Reductions of mortality and morbidity are the ultimate treatment goals in ST-elevation myocardial infarction (STEMI). Therefore, primary endpoints in clinical studies of new therapeutic interventions are ideally events relevant to patients such as death, reinfarction or new symptoms of heart failure. However, studies with clinical endpoints are associated with several shortcomings. The incidence of the event of interest (e.g. death following myocardial infarction) is increasingly low during short- or medium-term follow-up given the advances in treatment. Furthermore, some events might not be linked to atherosclerotic disease resulting in low sensitivity. As a consequence, large sample sizes and long follow-up periods are required absorbing time and financial resources. Missing data and noncompliance are also more likely in longer-lasting studies [1].

Surrogate endpoints can overcome some of these problems allowing for a reduction in sample size and follow-up duration as well as studying pathophysiological mechanisms of disease thereby improving trial efficiency. Surrogate endpoints are alternative endpoints "used as a substitute for a clinically meaningful endpoint that measures directly how a patient feels, functions or survives. Changes induced by a therapy on a surrogate endpoint are expected to reflect changes in a clinically meaningful endpoint" [2]. As compared to true clinical endpoints, surrogate endpoints should ideally be easy to measure and should occur more frequently and earlier in the course of the disease.

Cardiac magnetic resonance (CMR) imaging offers a variety of parameters potentially suited as surrogate endpoints and is increasingly being used in clinical trials of STEMI. Following a short conceptual overview of surrogate endpoints, we describe several of these CMR parameters and their value in infarction trials.

### Surrogate Endpoints

#### Definition

According to a widely known definition published by a working group of the National Institutes of Health, a surrogate endpoint is "a biomarker that is intended to substitute for a clinical endpoint. A surrogate endpoint is expected to predict clinical benefit (or harm or lack of benefit or harm) based on epidemiologic, therapeutic, pathophysiologic, or other scientific evidence." [3]. A biological marker is defined as "a characteristic that is objectively measured and evaluated as an indicator of normal biological processes, pathogenetic processes, or pharmacologic responses to a therapeutic intervention" [3]. Surrogate endpoints are thus a subset of biological markers.

#### Validity

According to the "International Conference on Harmonisation (ICH)" the strength of the evidence for surrogacy depends upon (i) the biological plausibility of the relationship, (ii) the demonstration in epidemiological studies of the prognostic value of the surrogate for the clinical outcome and (iii) evidence from clinical trials that treatment effects on the surrogate correspond to effects on the clinical outcome [4]. The simple biological association between surrogate and clinical outcome is therefore not sufficient for a marker to qualify as a surrogate endpoint.

#### Reliability

Apart from validity, surrogate markers must also prove a high degree of reliability. Reliability refers to the consistency of measurements with only minimal variability. Reliability may be estimated through a variety of methods such as intraobserver repeatability (degree of variability if measurements of the surrogate are repeated under identical circumstances by the same person) or interobserver repeatability (degree of variability when measurements are repeated by a different observer). Reliability does not imply validity.

#### Limitations of Surrogate Endpoints

The use of surrogate endpoints is controversial. Most importantly, surrogate endpoints are often criticized for poor validity or even a complete lack of validation studies. Another point of criticism of surrogate endpoints relates to the safety of therapeutic interventions. Since surrogate endpoint studies usually enroll fewer patients than trials with clinical endpoints (indeed this is considered one of the essential advantages of surrogate trials), there is a substantial risk to underdiagnose rare adverse events.

### Cardiac Magnetic Resonance Imaging Parameters Potentially Suited as Surrogate Endpoints

#### Infarct Size

##### Basic Description

**Figure 1:**
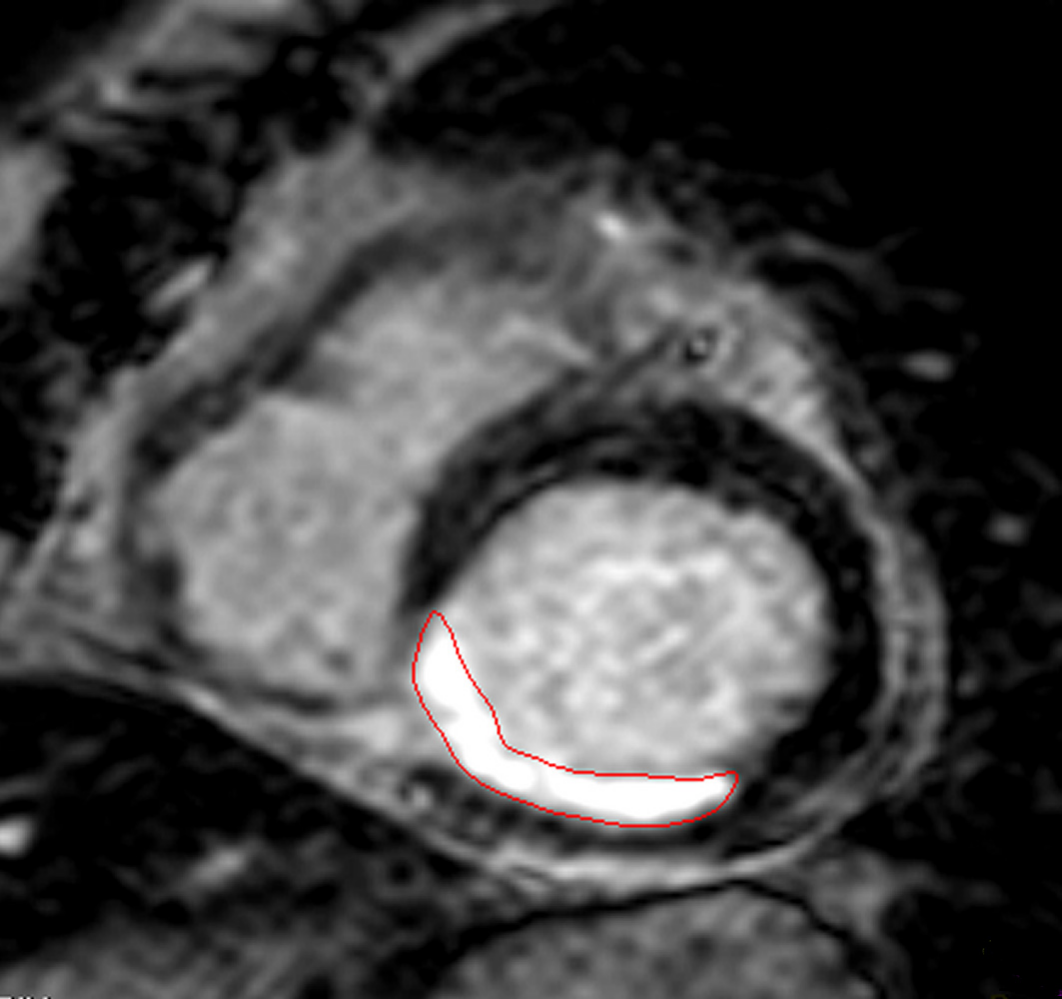
**Late enhancement CMR imaging showing infarcted myocardium (red contours) in a patient with inferior/inferoseptal STEMI due to occluded right coronary artery**.

Myocardial infarction can be visualised and quantified using inversion recovery imaging 10 to 15 minutes after intravenous administration of gadolinium contrast (late enhancement imaging). With correct settings, the area of infarcted myocardium appears bright whereas normal myocardium appears black (Figure 1). Experimental models have shown excellent agreement between the size and shape of late contrast enhancement in CMR and areas of myocardial necrosis or scar by histopathology [5,6].

In the first days following myocardial infarction, infarct volume is usually greatest possibly in part due to marked tissue swelling [7,8]. As necrotic tissue is replaced by scar, infarct size decreases over the course of several weeks (most pronounced in the first week) [7,8]. These remodelling processes are usually completed after 6 to 8 weeks and infarct size is stable thereafter [7,8]. The dynamic evolution of infarct volume following infarction must be taken into account when using infarct size as a surrogate endpoint in clinical trials. When measuring infarct size in the first days or weeks after infarction, it is important to adhere to a consistent time interval between infarction and CMR image acquisition across all patients whenever possible. Otherwise infarct size variability may be explained simply by the differences in timing of CMR assessment following infarction. Although the time from symptom onset to image acquisition could be used to define the interval, time from revascularization to image acquisition might be more appropriate since reperfusion injury can exert a major influence on infarct size (and subsequent myocardial salvage) and microvascular obstruction. With the latter approach, it stands to reason that the variability of differences in the time from symptom onset to reperfusion is a potential confounder and study sample size must be adapted accordingly. In the chronic phase the imaging time frame can be chosen more liberally. Advantages of performing CMR assessment early after infarction include the concomitant assessment of microvascular obstruction or the area at risk. In general, the decision when to measure infarct size must be based on trial specifics. A limitation of infarct size assessment by late enhancement CMR is the lack of standardized protocols for primary image acquisition (e.g. with regards to pulse sequences or dose of contrast agent). Recommendations for standardization have recently been published [9]. Newer phase-sensitive inversion recovery sequences are able to achieve a more consistent contrast between infarcted and normal myocardium which in turn might influence measurement variability in image analysis [10].

It should be noted that there are yet no data on the reliability of infarct size measurements between scanners from different vendors. This, however, would be a prerequisite for infarct size to qualify as a reliable endpoint in multicenter trials with a variety of scanners.

##### Image Analysis

For research purposes, quantitative analysis of infarct volume is best performed by delineating infarct borders in a stack of short-axis slices covering the whole ventricle. Infarct size can be expressed as absolute mass or percent of left ventricular mass (mass [grams] = volume [mL] × myocardial density [1.05 grams/mL]) [11].

Delineation of the infarct region can be performed by manual planimetry based on visual assessment of infarct borders or by using semiautomatic analysis software. Although manual tracing might be partially subjective, it has been used extensively by many CMR centers [12-14]. In an attempt to overcome the subjective nature of visual assessment and manual planimetry, several semiautomatic methods have been proposed [15]. Semiautomatic methods are based on enhanced signal intensity of the infarcted area as compared to normal myocardium. Infarcted myocardium can be defined by exceeding a threshold value of signal intensity as compared to a reference region located in healthy myocardium. Initial ex vivo studies suggested an image intensity threshold of 2 to 3 standard deviations above remote normal myocardium for infarct characterization [5,16]. However, spatial resolution for in vivo examinations is much lower mainly due to cardiac motion. Therefore, a threshold value of 5 standard deviations might be more appropriate in the clinical setting [12]. However, there is currently no consensus which threshold is best/preferable for infarct size assessment. In principle, choosing a lower threshold value such as 2 standard deviations will likely include the border zone of the infarct possibly leading to overestimation of infarct size. With a higher cut-off value such as 5 standard deviations only areas with high signal intensities like the core will be characterized as infarcted. Naturally, infarct characterization is also highly dependent on the choice (signal intensity) of the remote region. Some of these problems can be avoided using the full-width at half-maximum method. A region in the central infarct core is chosen as reference [17]. Myocardium displaying a signal intensity of at least 50% of the reference region will be marked as infarcted. Full-width at half-maximum might be inaccurate in homogeneously gray infarcts without a clear hyperintense core or in infarcts with a patchy necrosis pattern [15]. Of note, the newer semiautomatic methods require a certain degree of subjective input as well. Endocardial and epicardial borders must still be drawn manually. This includes the endocardial infarct border which can comprise up to 50% of the infarct perimeter [15]. Furthermore, artefacts and obvious misclassifications of healthy tissue as myocardial infarction can be manually corrected. Semiautomatic methods have so far been tested in few patients only [15]. Flett et al. compared the reproducibility of 7 late enhancement quantification techniques in 20 patients with acute myocardial infarction: Manual quantification, thresholding by 2, 3, 4, 5, or 6 standard deviations above remote myocardium, and the full-width at half-maximum technique [18]. The full-width at half-maximum technique was the most reproducible compared with any other method. Semiautomatic methods are constantly being refined and more complex image analysis algorithms will likely lead to further improvements [19-21].

Apart from quantitative analysis of infarct volume as described above, late enhancement imaging can also be used to measure the extent of infarct transmurality which provides additional information in predicting improvement in contractile function after myocardial infarction [22].

##### Validity and reliability

When applying the above-mentioned ICH criteria, validity for infarct size as a surrogate endpoint is relatively high: The capacity of infarct size to predict various clinical endpoints has been demonstrated in several epidemiological studies [23,24]. It has been reported that infarct size measurement by CMR is a stronger predictor of outcome than left ventricular function and volumes [24]. Also, the relationship between the surrogate infarct size and clinical outcome seems biologically plausible. However, evidence from clinical trials that treatment effects on the surrogate also correspond to effects on the clinical outcome is more difficult to establish. An example of a sequential approach with a positive surrogate endpoint study subsequently triggering a trial with true clinical endpoints has recently been presented [25,26]. In a randomized controlled trial of 144 patients with STEMI, intracoronary bolus administration of abciximab led to significant reductions in CMR infarct size as compared to standard intravenous bolus application (presumably through higher local drug concentrations that can be achieved through the intracoronary route) [25]. Based on these favorable effects on a surrogate endpoint, a large study (1912 patients) with a primary clinical endpoint of mortality, reinfarction and new heart failure symptoms has been initiated [26].

For manual tracing, infarct size measurement shows excellent interobserver reliability in the acute and chronic setting [14].

##### Comparison to alternative methods

Infarct size assessment by CMR offers several advantages over alternative methods. Owing to its high spatial resolution, it is possible to detect and quantify small endocardial infarcts which are often missed by single photon emission computed tomography (SPECT) imaging [6,27]. Given the high efficacy of modern reperfusion therapy with infarct size ≤ 10% of left ventricular mass in almost one half of patients, this aspect is of great importance [28]. It must be emphasized that the ability of CMR to detect smaller infarcts and therefore potentially smaller differences in infarct size between treatment groups does not necessarily translate into a reduced sample size for a given trial unless the standard deviation of measurements is also reduced. Apart from alpha (usually set at 0.05) and the desired power (usually set at 0.80 or 0.90), sample size for a standard two-arm superiority trial is dependent on the expected difference between treatment arms (the smaller the difference to be detected, the more patients will have to be enrolled) and its standard deviation. Patient-to-patient variability (reflected by the standard deviation) is naturally dependent on built-in differences in infarct size, but also on extraneous variability such as inconsistent data acquisition or image analysis technique. Thus far, the (limited) literature does not support any reduction in the overall standard deviation of CMR infarct size measurements compared to SPECT [29].

Furthermore, SPECT imaging is associated with exposure to ionizing radiation which might become especially relevant in longitudinal studies with multiple assessments of myocardial morphology and function. It should, however, be kept in mind that most patients with myocardial infarction are relatively old and longevity will likely be limited by cardiovascular morbidity rather than the risk imposed by additional radiation.

Infarct size may also be quantified through cardiac enzyme analysis by estimating the cumulative „area under the curve" or peak enzyme release in serial measurements [30]. However, CMR offers the advantage of obtaining additional parameters such as left ventricular volumes, ejection fraction, microvascular obstruction, myocardial salvage or infarct-associated complications. This aspect might be especially important in interpreting study results in their pathophysiological context and generating hypotheses for mechanisms of action for the therapeutic intervention under examination. In contrast to enzymatic analysis, CMR can also be used for serial assessments to evaluate postinfarction remodelling.

#### Myocardial Salvage

##### Basic Description

**Figure 2:**
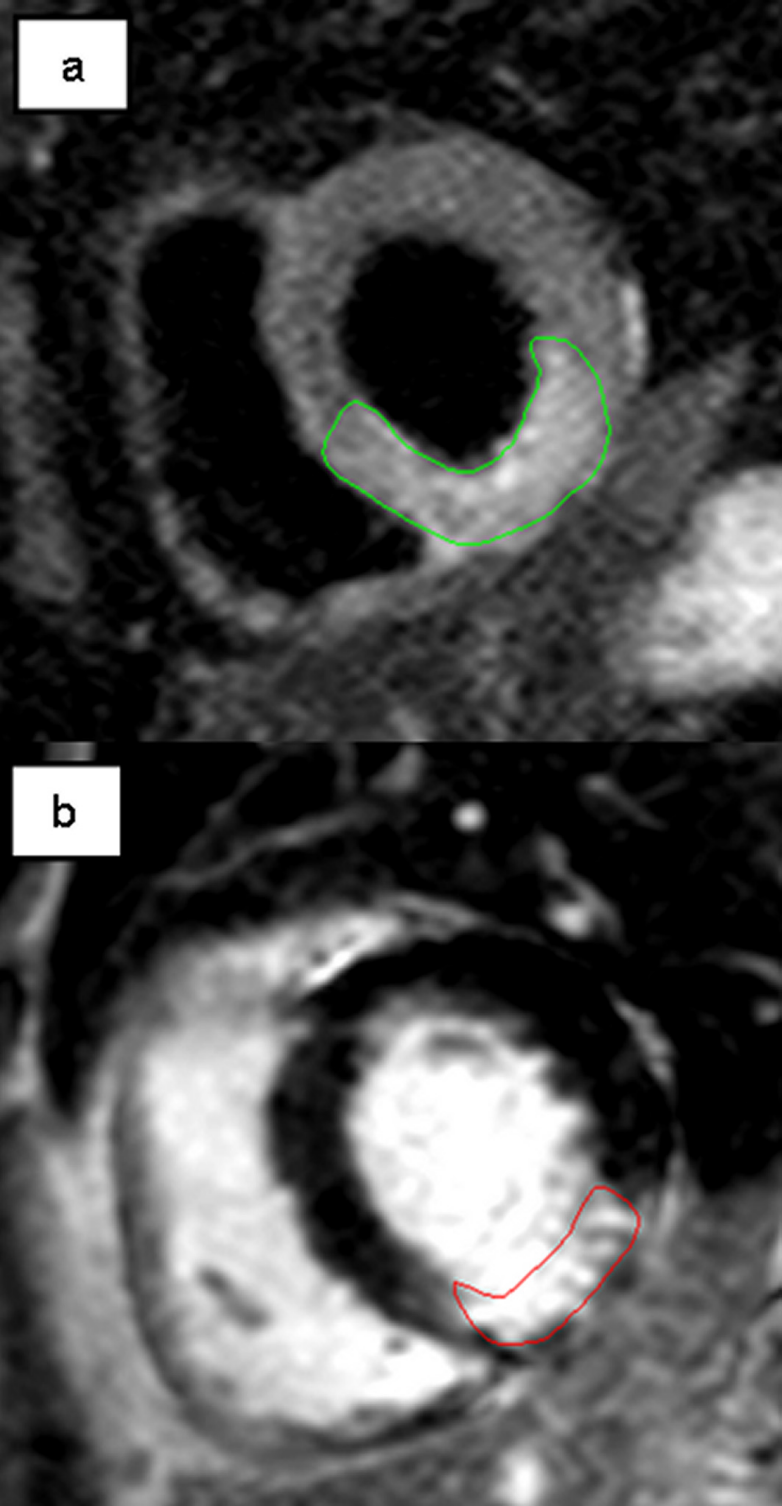
**Patient with inferior/inferolateral STEMI due to subtotal stenosis of right coronary artery**. a. T2-weighted image acquisition for the detection of myocardial edema (green contours), corresponding to the area at risk. b. Late enhancement CMR imaging showing infarcted area (red contours). Myocardial salvage can be calculated by comparing the area at risk in T2-weighted and infarct size in late enhancement images.

Myocardial salvage, which is defined as salvaged tissue following reperfusion therapy, holds promise as a surrogate endpoint. The area of high signal (edematous myocardium) on T2-weighted CMR imaging likely reflects the area at risk in acute myocardial infarction (Figure 2a) [31]. By comparing the area at risk in T2-weighted and final infarct size in late enhancement CMR images, the proportion of myocardial salvage can be assessed retrospectively (Figure 2a + b) [32]. In myocardial salvage assessment, reduction of infarct size can also be considered the main biological target, however with a „built-in" adjustment for the area at risk. Therefore, many of the characteristics for infarct size mentioned above are also true for the assessment of myocardial salvage. Theoretically, there are advantages of measuring salvaged myocardium over infarct size as an indicator of therapeutic efficacy in clinical trials. To illustrate this point consider a two-arm randomized trial of a novel therapeutic intervention with baseline imbalances of anterior and non-anterior infarctions between groups and subsequent differences in the area at risk. Since the area at risk can by itself explain over 50% of infarct size variability, it is likely that this constellation leads to differences in final infarct size between groups independent of the therapeutic intervention under examination [33]. Small differences in the area at risk may result in significant variation of infarct size, underscoring the fact that most of infarct size variability is due to the extent of the myocardium at risk [33,34]. Therefore, measuring only infarct size might impose a potential bias and myocardial salvage may be a better surrogate endpoint than infarct size. This is especially true in non-randomized study designs or smaller randomized trials where imbalances of baseline characteristics between treatment groups are frequent. In large randomized trials baseline imbalances are less likely, however at the cost of increased sample size.

As for infarct size, there is a lack of standardization with regard to image acquisition and analysis. Also, in the setting of multicenter trials, the potential variability of infarct size measurements between scanners from different vendors should be taken into account. Currently, most of the clinical experience in visualising myocardial edema has been obtained using inversion recovery turbo spin echo (TSE) sequences with suppression pulses for both fat and blood. Although these sequences provide useful images in most clinical cases, they have inherent limitations such as artefact susceptibility, variability in signal intensity if phased array coils are used and low contrast-to-noise ratio [35]. Slow flowing blood in particular near dyskinetic segments in the apex and between trabeculae may not be suppressed sufficiently. The corresponding high signal can, therefore, erroneously be included in the delineation of the area at risk [35]. One method to reduce this in clinical practice is to compare T2-weighted images of the same cardiac phase side-by-side with cine images to verify wall thickness. Newer sequences might account for several of these limitations. Specifically, a T2-prepared steady state free precession (SSFP) technique has been studied as an alternative to conventional T2-weighted TSE imaging [36]. In a trial of 31 patients with myocardial infarction (22 acute, 9 chronic) T2-prepared SSFP provided images with fewer artefacts and better diagnostic accuracy compared to T2-weighted TSE imaging albeit at reduced signal-to-noise ratio [36]. A hybrid method of the aforementioned SSFP sequence and conventional T2-weighted TSE imaging has also been presented combining the advantages of SSFP imaging with the higher signal-to-noise and contrast-to-noise ratio of T2-weighted TSE imaging [37]. An alternative approach to T2-weighted imaging using signal intensity as a surrogate for T2 prolongation is the direct determination of myocardial T2 relaxation times. Thereby, several limitations associated with conventional T2-weighted imaging can be addressed, resulting in a potentially more reliable method for the detection of myocardial edema and the area at risk [38]. While these new developments hold great promise, they have so far been studied in few patients only.

At present, there are only limited data with regard to the natural evolution of postinfarction edema by CMR. In a canine model, edema could be detected by CMR shortly after coronary occlusion [39]. In a small study of patients with STEMI, edema was not significantly different between day 1 and 1 week after infarction [40]. It is unclear how long edema persists following myocardial infarction. In patients with hypertrophic obstructive cardiomyopathy undergoing septal artery embolization, edema could be found after 28 days in all patients, whereas it was no longer present after 3 months [41]. Other studies in acute reperfused ST-elevation myocardial infarction patients have shown long-lasting postinfarction edema up to 12 months [42,43].

##### Image Analysis

Most of the techniques described for the measurement of infarct size also apply to edema assessment. However, due to the aforementioned limitations of current T2 sequences for edema assessment, image analysis can be challenging in some patients. Image interpretation depends on regional differences in myocardial signal intensity and the purely visual delineation of edema borders offers the potential for subjective error. Cut-off values for defining abnormal vs. normal tissue for semiautomated quantification are not sufficiently standardized. A quantitative T2 mapping technique has recently been introduced which offers the potential for increased accuracy in image analysis [38].

##### Validity and reliability

SPECT myocardial salvage has been successfully used as a surrogate endpoint in several clinical trials [44-46] and the first studies using myocardial salvage by CMR as a primary endpoint have been published [47,48]. Recently, a study showed that the amount of myocardial salvage assessed by CMR also predicts patient outcome [49]. The prognostic value of the salvage area at risk is consistent with previous SPECT studies [50]. However, as with infarct size, there are yet no clear data demonstrating that specific therapies able to increase myocardial salvage can also favorably influence patient-relevant endpoints. Recently, acceptable reliability for myocardial salvage assessment has been shown [51].

##### Comparison to alternative methods

Myocardial salvage can also be assessed by SPECT [44,46]. However, CMR has several advantages over SPECT. As mentioned above, CMR yields higher spatial resolution which allows the assessment of small subendocardial infarcts often elusive to SPECT imaging [6,27]. Furthermore, CMR can assess the salvaged myocardium retrospectively a few days after infarction and does therefore not interfere with acute patient management (in SPECT imaging the radioactive tracer must be injected before recanalization of the infarct-related artery in the emergency department setting). Salvage assessment by CMR can also be performed with a single examination whereas in SPECT it is necessary to perform two subsequent measurements for assessment of the initial perfusion defect and final infarct size. Finally, CMR avoids radiation exposure. CMR may therefore be superior to assess myocardial salvage.

#### Microvascular Obstruction

##### Basic Description

**Figure 3:**
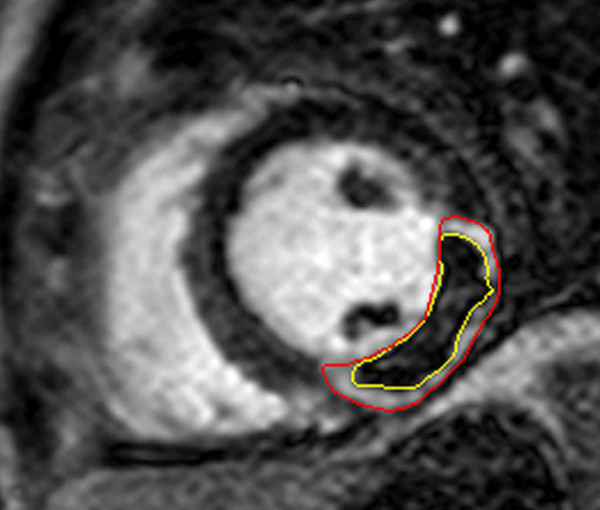
**Late enhancement CMR imaging showing infarcted myocardium (red contours) in a patient with lateral STEMI due to occluded left circumflex artery**. The central hypointense core within the infarct represents microvascular obstruction (yellow contours).

Early recanalization of the infarct-related artery is the primary treatment target in the first hours after symptom onset in acute STEMI [52]. However, restoration of epicardial flow does not necessarily translate into adequate perfusion of the microcirculation. Areas of impaired microcirculatory flow can be directly visualised and quantified by CMR as microvascular obstruction. Following contrast administration infarcted zones take up gadolinium and subsequently appear bright. However, in areas of severely compromized perfusion contrast take-up is absent. Areas of microvascular obstruction can therefore be visualised as dark areas within the bright infarct (Figure 3).

Several methods for the assessment of microvascular obstruction by CMR have been proposed [53]. Image acquisition during first myocardial pass of gadolinium, early imaging in the first minutes after contrast administration and late imaging approximately 15 minutes after contrast injection. The extent of microvascular obstruction gradually declines between first-pass and late imaging. The observed differences over time reflect the persistent slow diffusion of contrast or collateral filling into areas with a less compromized microcirculation. These regions subsequently display smaller or completely absent zones of hypoenhancement on late imaging. Microvascular obstruction on late imaging therefore likely reflects areas of a more severely disturbed microcirculation whereas microvascular obstruction on early imaging is more sensitive for the detection of only small or less impaired areas of microvascular injury. At present, there is no consensus which of these slightly differing techniques to apply. However, in the largest patient series to date late image acquisition (approximately 15 minutes after contrast administration) was superior to early image acquisition (approximately 1 minute post contrast administration) in predicting clinical outcome [54]. Myocardial regions displaying delayed, yet not fully absent perfusion might therefore be of only minor importance for clinical prognosis.

Given the time dependency of presence and extent of microvascular obstruction on the time between contrast administration and image acquisition, it is important to adhere to strict methodology within the clinical trial setting.

##### Image Analysis

Quantitative analysis of microvascular obstruction within the infarct zone is performed in a stack of short-axis slices using either manual planimetry or semiautomatic methods. Techniques are similar to those described above for the assessment of infarct size.

##### Validity and reliability

Microvascular obstruction is reasonably valid to be used as a surrogate endpoint in clinical trials. Numerous studies relating to pathophysiological mechanisms have been published [55] and the association of CMR microcirculatory injury and adverse clinical prognosis is well established [54,56,57]. However, as with infarct size and myocardial salvage, proof is lacking that therapeutic measures able to reduce markers of microvascular obstruction can also favorably influence true clinical endpoints [55]. There are yet no published reliability studies for the assessment of microvascular obstruction.

##### Comparison to alternative methods

Several other modalities are available for detecting microvascular obstruction such as myocardial blush grading on invasive angiography, evaluation of electrocardiographic ST-resolution or myocardial contrast echocardiography. Microvascular obstruction assessed by CMR might be superior to myocardial blush grading and ST-resolution in predicting functional recovery after myocardial infarction [58,59]. In contrast to myocardial blush grading, CMR image acquisition for the assessment of microvascular obstruction is not performed immediately following coronary intervention. This might be advantageous since microvascular obstruction often expands during the first hours following reperfusion. Therefore, very early measurement might not reflect the true quantitative extent [60].

#### Left ventricular ejection fraction and volumes

##### Basic Description

CMR measures ventricular volumes and mass using a simple acquisition of a 3-dimensional stack of contiguous short-axis cines with full biventricular coverage. Currently, the standard technique to measure left ventricular ejection fraction and volumes is a breath-hold, SSFP sequence that provides optimal contrast between blood and myocardium. Current cine sequences use retrospective electrocardiographic gating, although prospective gating may be required in patients with irregular heart rhythm. A portion of the data is recorded during each cardiac cycle and data from several heart beats are then fused to form a continuous cine movie loop.

##### Image Analysis

Calculation of left ventricular ejection fraction and volumes is commonly performed in short axis images following planimetry of endocardial and epicardial contours. The borders are typically traced at enddiastole and endsystole.

##### Validity and reliability

Left ventricular ejection fraction and volumes are important predictors for survival after acute myocardial infarction [61,62]. CMR is an accurate and highly reproducible technique for measuring left ventricular ejection fraction and volumes and is thus well suited to assess postinfarction remodelling through serial assessment of left ventricular function and morphology [63].

##### Comparison to alternative methods

Echocardiography and gated SPECT are widely available techniques available for measuring cardiac function and volumes. Gated SPECT suffers from relatively low spatial resolution. Compared to echocardiography, CMR has been shown to be significantly more accurate with less interobserver and intraobserver variability as well as superior reproducibility [64]. Notably, CMR assessment of the aforementioned parameters is less dependent on geometric assumptions. This aspect is especially important in patients after myocardial infarction where regional alterations of left ventricular geometry are frequent (e.g. aneurysms). Consequently, CMR is the technique of choice for longitudinal studies of left ventricular ejection fraction or remodelling over time.

#### Other CMR parameters

##### Intramyocardial Hemorrhage

**Figure 4:**
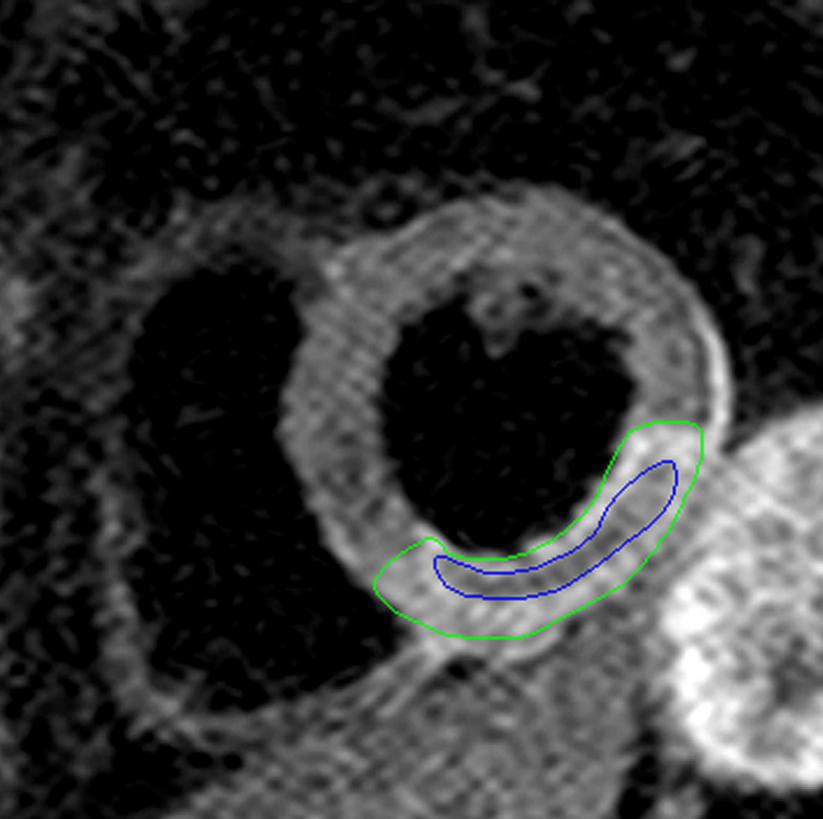
**T2-weighted spin echo imaging in a patient with inferior/inferolateral STEMI due to occluded right coronary artery showing myocardial edema (green contours)**. The hypointense zones within the edematous region likely represent intramyocardial hemorrhage (blue contours).

A subset of patients with acute myocardial infarction display hypointense zones within the area at risk in T2-weighted spin echo sequences (Figure 4) [65]. These regions likely correspond to intramyocardial hemorrhage and are associated with adverse remodelling of the left ventricle [65]. T2*-weighted gradient echo sequences are also able to visualise hemorrhagic infarcts and might be more sensitive to the susceptibility effects of hemorrhage than spin-echo imaging [66]. The presence of such hemorrhage is associated with microvascular obstruction and has been shown to be a strong predictor of adverse remodelling after infarction [65,67]. However, the clinical significance of intramyocardial hemorrhage on hard clinical outcome has not yet been established. In conclusion, further validation and reliability studies are necessary for this parameter to qualify as a surrogate endpoint in clinical trials.

##### Infarct core and border zone

The infarct region can be further subclassified into core and border zone depending on relative signal intensity as compared to normal myocardium (semiautomatic analysis). In one trial the infarct core has been defined by a signal intensity ≥ 3 standard deviations of remote normal myocardium whereas the peri-infarct border zone was classified by a signal intensity between 2 and 3 standard deviations [68]. The border zone represents a mixture of healthy and structurally damaged myocytes and might be a substrate of ventricular arrhythmia [69]. The topic has so far been studied in only few patients in the chronic phase after infarction.

### General limitations of post-infarct CMR imaging

Important limitations of CMR imaging in post-infarct patients are contraindications such as pacemakers, internal defibrillators, claustrophobia or hemodynamic and electrical instability. Also, there are concerns about the use of gadolinium based contrast agents in patients with advanced renal failure due to the risk of developing nephrogenic systemic fibrosis [70]. When planning a trial in myocardial infarction with CMR endpoints, one should be aware that these factors are often defined as exclusion criteria. Even in the cohort finally randomized, 5-10% of patients will eventually not undergo CMR examination for various reasons [25,48]. Furthermore, in patients with atrial fibrillation or significant ventricular ectopy, there can be degradation in image quality.

### Randomized controlled STEMI trials with primary CMR endpoints

Table 1 summarizes published randomized controlled trials for the treatment of myocardial infarction in the acute setting using CMR parameters as a primary study endpoint (restricted to trials published until December 2010 and listed in MEDLINE). Trials with a non-CMR primary endpoint or those not defining the primary endpoint were excluded. Numerous further studies are currently being conducted.

**Table 1:** Randomized controlled trials for the treatment of myocardial infarction in the acute setting using CMR parameters as a primary study endpoint (sorted by date of publication)

Primary CMR endpoint	Study	Treatment	Number of patients	Year published
Myocardial salvage index at days 2-4	Thiele et al. (LIPSIA-N-ACC study)[48]	High-dose N-acetylcysteine	251	2010
LV endsystolic volume index at 10-14 weeks	Abbate et al.[71]	Interleukin-1 blockade with anakinra	10	2010
LV ejection fraction at 6 months	Wöhrle et al.[72]	Autologous intracoronary bone-marrow cell therapy	42	2010
Myocardial salvage at 3 months	Lonborg et al.[47]	Ischemic postconditioning	118	2010
Infarct size at 4-6 months	Haeck et al.[73]	Proximal embolic protection and thrombus aspiration	206	2010
Infarct size and LV ejection fraction at 90 days	Patel et al. (APEX-AMI trial)[74]	Pexelizumab (anti-C5 complement antibody)	99	2010
LV endsystolic volume index at 24 weeks	Weir et al.[75]	Eplerenone	100	2009
LV ejection fraction at 6 months	Tendera et al. (REGENT study)[76]	Intracoronary infusion of bone-marrow derived selected CD34+CXCR4+ cells versus non-selected mononuclear cells	200	2009
Infarct size at 1 month	Song et al.[77]	Upstream high-dose tirofiban treatment		2009
LV ejection fraction at 4 and 12 months	Dill et al.[78]	Intracoronary administration of bone-marrow derived progenitor cells	54	2009
Infarct size after 5 days	Atar et al. (FIRE study)[79]	FX06	234	2009
Infarct size and microvascular obstruction at 2 days	Thiele et al.[25]	Intracoronary versus intravenous bolus abciximab application	144	2008
Infarct size at 3 days	Hahn et al.[80]	Distal protection device	39	2007
Global and regional myocardial function at 3 months	Engelmann et al.[81]	Granulocyte colony-stimulating factor	44	2006
LV ejection fraction at 6 months	Kang et al. (MAGIC Cell-3-DES trial)[82]	Intracoronary infusion of mobilized peripheral blood stem cells	96	2006
Systolic wall thickening at 6 months	Ripa et al. (STEMMI trial)[83]	Granulocyte colony-stimulating factor	78	2006
LV ejection fraction at 4 months	Janssens[84]	Transfer of autologous bone-marrow derived stem cells in the infarct-related artery	67	2006
Infarct size at 6 months	Thiele et al.[85]	Pre-hospital combination-fibrinolysis plus conventional care versus pre-hospital combination-fibrinolysis plus facilitated percutaneous coronary intervention	164	2005
LV ejection fraction at 6 months	Wollert et al. (BOOST trial)[86]	Intracoronary transfer of autologous bone-marrow cells	60	2004
LV volumes at 6 months	Darasz et al.[87]	Captopril and xamoterol	70	1995

Abbreviations: CMR = cardiac magnetic resonance; LV = left ventricular.

## Summary and conclusions

CMR is a safe technique, even in the first days after infarction and offers a variety of established and novel parameters potentially suitable as surrogate endpoints in clinical trials of STEMI. It allows the evaluation of function, infarct extent, salvaged myocardium and microvascular obstruction, and can be acquired easily within 30 to 40 minutes. The choice of marker is naturally dependent on the specific question of the trial to be conducted. However, choosing the most appropriate surrogate endpoint for the question at hand cannot restrict itself to a biologically plausible association between surrogate and clinical outcome. Other qualities of validity must also be demonstrated. Furthermore, surrogate endpoints should demonstrate high measurement reliability which can be considered a specific strength of most CMR parameters. Definite proof of validity is more difficult to establish. Therefore, the use of CMR surrogate endpoints in trials of myocardial infarction mandates a thoughtful interpretation of study results. It seems reasonable to use CMR surrogate endpoint studies mainly to prove fundamental biological activity and to evaluate pathophysiological mechanisms of novel therapeutic interventions. This can ultimately guide the decision whether to conduct larger studies with endpoints truly relevant to patients. Further studies should focus to address some of the limitations of CMR endpoints in myocardial infarction.

## List of abbreviations

STEMI: ST-elevation myocardial infarction; CMR: Cardiac magnetic resonance; ICH: International Conference on Harmonisation; SPECT: Single photon emission computed tomography; TSE: Turbo spin echo; SSFP: steady state free precession.

## Competing interests

The authors declare that they have no competing interests.

## Authors' contributions

SD drafted the initial version of the manuscript. All authors made substantial contributions in critically revising the manuscript for important intellectual content, read and approved the final version.
